# Using environmental DNA analyses to assess the occurrence and abundance of the endangered amphidromous fish *Plecoglossus altivelis ryukyuensis*

**DOI:** 10.3897/BDJ.8.e39679

**Published:** 2020-01-14

**Authors:** Yoshihisa Akamatsu, Gen Kume, Masuji Gotou, Takanori Kono, Takuma Fujii, Ryutei Inui, Yoshihisa Kurita

**Affiliations:** 1 Yamaguchi University, Ube, Japan Yamaguchi University Ube Japan; 2 Kagoshima University, Kagoshima, Japan Kagoshima University Kagoshima Japan; 3 Kagoshima University, Amami, Japan Kagoshima University Amami Japan; 4 Fukuoka Institute of Technology, Fukuoka, Japan Fukuoka Institute of Technology Fukuoka Japan; 5 Kyushu University, Fukutsu, Japan Kyushu University Fukutsu Japan

**Keywords:** Monitoring methods, threatened species, stock management, conservation, qPCR

## Abstract

The Ryukyu ayu *Plecoglossus altivelis ryukyuensis* is an endangered amphidromous fish that inhabits rivers in the Ryukyu Archipelago (Japan). Populations of the species have declined dramatically. Consequently, the Ryukyu ayu has been registered as a natural monument in Japan and monitoring surveys with direct catching are restricted legally. This restriction, unfortunately, makes monitoring of population abundances difficult and creates a barrier to both advancing understanding of the species’ status and the development of appropriate conservation plans.

We developed a non-invasive monitoring methodology using eDNA analyses. We designed a specific quantitative PCR assay for the Ryukyu ayu using the mitochondrial ND4 region. Using this primer/probe set, we conducted eDNA analyses in three rivers on Amami-Ohshima Island. The DNA fragments were amplified from the eDNA extracted from natural water in each river. The numbers of DNA fragments detected were positively correlated with individual counts of fish obtained by visual snorkelling surveys. Our method does not contravene restrictions and facilitates abundance monitoring of this endangered fish species.

## Introduction

Monitoring the density and distributions of populations is an essential component of the conservation of endangered species (Cobos and Alonso Bosch 2018, Boon et al. 2019). Fish monitoring is usually based on data obtained from direct catches, electrofishing, trapping and visual observation. However, legal restrictions promulgated for the protection of endangered fishes often constrain investigations that use conventional ‘invasive’ methods, thereby reducing confidence in estimates of abundance and distribution. In these cases, less damaging methods should be investigated to improve ecological monitoring of protected species and evaluate the current state of populations.

The Ryukyu ayu *Plecoglossus altivelis ryukyuensis* is an endangered amphidromous fish that inhabits rivers in the Ryukyu Archipelago. The Ryukyu ayu has diverged genetically and morphologically from the related subspecies *Plecoglossus altivelis altivelis*, which occurs in more northern sections of the Japanese archipelago (other than Hokkaido; Ikeda et al. 2003). The Ryukyu ayu was originally found in the waters of Okinawa Island and Amami-Oshima Island. The Okinawa Island population became extinct in 1978. Thus, the Amami-Oshima Island population, which is the last wild population of Ryukyu ayu, is now classified as critically endangered (CR) in the Red List published by the Japanese Ministry of the Environment, Japan (2018). The local and national governments prohibit collection or injury of these fish.

Environmental DNA (eDNA) obtained from natural water samples provides valuable data for monitoring fish species. This non-invasive procedure has been successfully applied to several species in diverse aquatic systems (Thomsen and Willerslev 2015, Laramie et al. 2015, Atkinson et al. 2018). Multiple studies have shown that the probability of detecting fish using eDNA can be higher than that of conventional assessment methods, such as direct observation and capture (Dejean et al. 2012, Takahara et al. 2013). Additionally, eDNA analysis requires less sampling effort and can cost up to 67% less than conventional methods (Evans et al. 2017), although the reliability of abundance and biomass estimations of populations is still debatable (e.g. Buxton et al. 2017); this said, eDNA methods can be used to determine the presence or absence of a species. In addition, a recent study succeeded in using eDNA analyses to estimate the abundance/biomass of *P. a. altivelis* in a river (Doi et al. 2016). Regarding legally protected species, monitoring is usually conducted by visual surveys because of restrictions on direct catch methods. A procedure using only small volumes of water may be able to monitor the abundance and distribution of protected organisms without the difficulties of a visual survey (i.e. high turbidity and many hidden structures) or contravening legal regulations.

In this study, we developed species-specific primers and a probe for detecting the eDNA of the Ryukyu ayu via quantitative PCR (qPCR). We ground-truthed the relationship between eDNA concentrations and fish abundance through visual snorkelling surveys in the rivers studied. We were also able to investigate the spatial distribution of the Ryukyu ayu along the lengths of the rivers.

## Materials and method

### Field surveys

**Figure 1.**
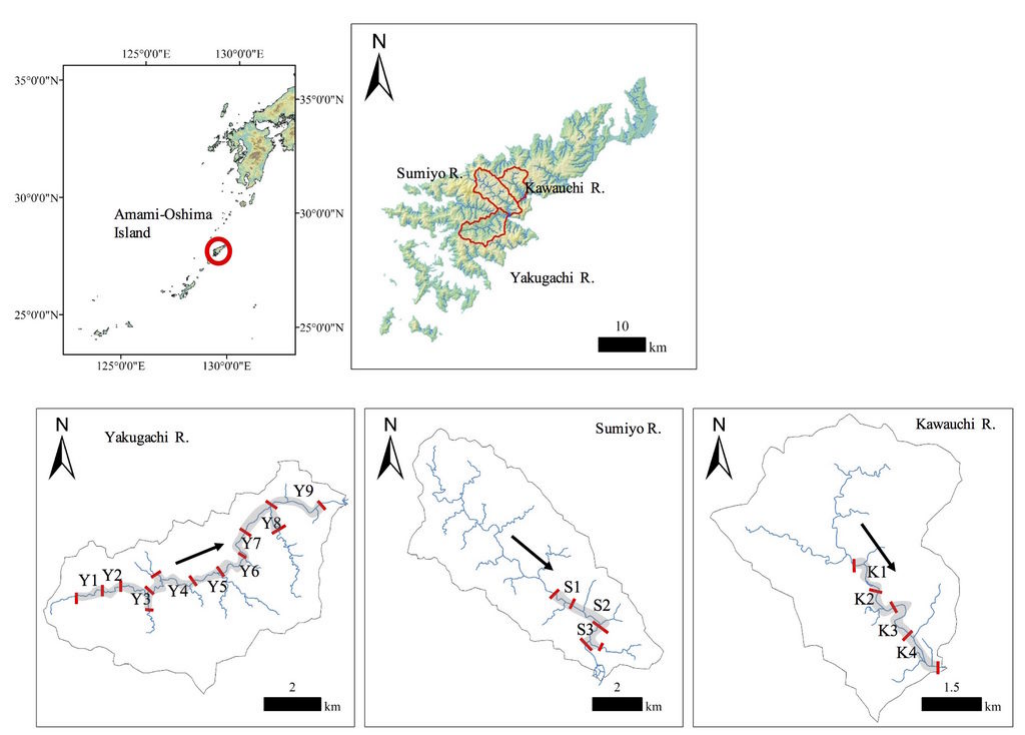
Maps of the study sites. Arrows indicate water flow direction in three rivers. Codes refer to segments of individual rivers in which surveys were conducted.

Field surveys were conducted in three rivers (the Yakugachi, Sumiyou and Kawauchi rivers) on Amami-Oshima Island, Japan (Fig. 1). The catchment areas and river lengths were as follows: Yakugachi River, 47.8 km^2^ and 15.1 km; Sumiyou River, 48.5 km^2^ and 15.5 km; Kawauchi River, 28.3 km^2^ and 12.3 km. All rivers drain into the sea on the east coast of Amami-Oshima Island. Ryukyu ayu individuals swim up these rivers every year to grow (February–May) and spawn (December–February). The hatching larvae flow down to sea.

**Visual surveys by snorkelling**

We counted the numbers of individual Ryukyu ayu in the Yakugachi River on 16 November 2017; numbers were counted in the Sumiyou and Kawauchi Rivers on 17 November 2017. The survey areas extended from the seaward end of the freshwater zone upstream to the uppermost reach of Ryukyu ayu distribution area in each of the rivers. We divided the survey areas in each river into segments of ca. 1 km length; thus, the Yakugachi River was divided into 12 segments and the other two rivers into four segments each (Fig. 1). Visual counts of the fish were made by snorkelling downstream through the length of each segment. The methodology of the visual survey followed Watanabe and Ito (1999). The visual surveys were started at 13:00 h on 16 November and at 10:00 h on 17 November. Each survey lasted 60–120 min.

**Field sampling for eDNA**

We collected 1 litre surface water samples for eDNA analyses in the shallows near the downstream end of each survey segment just after each snorkelling survey. Snorkelling survey and water sampling were conducted by different personnel. Each water sample was packed in a plastic bag containing benzalkonium chloride (eDNA preservative) at a final concentration of 0.01% and transported to the laboratory. Then, the water samples were filtered through GF/F glass fibre filters (pore size 0.7 µm, GE Healthcare, Japan) on the day of sampling and stored at –20°C. We incorporated an ‘equipment blank’ and ‘cooler blank’ as negative controls for each filtering and sampling step, respectively. As a cooler blank, we carried 1 litre of ultra-pure water in a bottle from our laboratory to the sampling field and it was treated identically to the sampled water bottles, except that it was not opened at the field sites. In the laboratory, we filtered the cooler blank and the equipment blank (1 litre DNA-free distilled water prepared in the laboratory) as negative controls after filtering the test samples on each sampling day.

### DNA extraction and qPCR

We extracted DNA from the filters with a DNeasy Blood & Tissue Kit (QIAGEN, Netherlands), following the procedures described by Doi et al. (2016).

We designed a new forward primer (Pa-ND4F: 5'‑ATAGCACTTCCACTGACAGCCACC‑3'), reverse primer (Pa-ND4R: 5'‑AGTAGGACCAGTTAAACATGGCCGTG‑3') and probe (5'‑FAM-GGTTTATTGCTAACCTAGCTAACCTGGC-TAMRA‑3'), based on the sequences of the mtDNA NADH dehydrogenase subunit 4 (ND4) region of the Ryukyu ayu, registered in GenBank (accession numbers AB181780–AB181799; www.ncbi.nlm.nih.gov/genbank). The primer and probes did not amplify extracted DNA of *Mallotus villosus* and *Hypomesus nipponensis*, species that belong to the same family as the Ryukyu ayu (Suppl. material 1 shows alignments of each primer/probe with osmeriform relatives). Both species do not co-occur on Amami-Ohshima Island, but are widely sold as food products.

The eDNA samples were quantified by real-time TaqMan" qPCR using the PikoReal Real-Time PCR System (Thermo Fisher Scientific). The qPCR procedure was optimised using extracted DNA from tissue samples of the Ryukyu ayu and conducted in 8 µl reaction volumes with 125 nM primer and probe, 4 µl TaqMan Environmental Master Mix 2.0 (Thermo Fisher Science, USA), uracil DNA glycosylase (Thermo Fisher Science) and 2 µl DNA template sample or 2 µl negative control samples (the cooler blank and the equipment blank as mentioned above). A dilution series of the synthetic linear DNA (124 bp: 5'‑ATAGCACTTC CACTGACAG CCACCTGGT GGTTTATTG CTAACCTAG CTAACCTGG CCCTCCCAC CTCTCCCCA ACCTTATGGG GGAGCTGGTC ATTATCACGG CCATGTTTAA CTGGTCCTACT‑3') (Takara) containing 2×10^1^, 2×10^2^, 2×10^3^, 2×10^4^ and 2×10^5^ copies per tube was also used in triplicate as the quantification standard in all qPCR assays. In addition, to avoid contamination, we performed the above qPCR set-up, including preparation and addition of the standards, in a separate room from that of the qPCR procedure. The thermal-cycling regime was as follows: 95°C for 3 min, followed by 55 cycles of 95°C for 10 s and 60°C for 20 s. We performed four replicates for each sample in the qPCR assay. The limit of detection (LOD) of the qPCR was one copy per reaction with four replicates. We analysed the qPCR results using the PikoReal software ver. 2.2.248.601 (Thermo Fisher Scientific). The species related to Ryukyu ayu, which are possibly recognised by our primer/probe set, are not distributed in the study area. We sequenced 8 qPCR products, to confirm that qPCR products, amplified from eDNA, were the target sequences of the Ryukyu ayu. Sequencing was carried out by an external agency (FASMAC, Japan). We examined the relationship between the eDNA concentration and individual numbers of the Ryukyu ayu using single regression analyses.

## Results

### Visual survey by snorkelling

Table 1 lists the numbers of individual fish counted in each river survey segment. The Ryukyu ayu were observed in most survey segments of the three rivers. We counted 187.6 ± 245.0 individuals (mean ± SD) in each survey segment and a total of 1688 individuals in the Yakugachi River. The numbers were 183.3 ± 317.5 and 550 individuals in the Sumiyou River and 350.5 ± 259.9 and 1402 individuals in the Kawauchi River, respectively. The highest density of individuals was counted in segment K3 (Fig. 1) in the Kawauchi River (732 individuals). No fish of this species were found in segment Y3 in the Yakugachi River or in segments S1 and S2 in the Sumiyou River.

### Optimising Ryukyu ayu eDNA detection to estimate fish abundance

The primer sets amplified the Ryukyu ayu DNA extracted from the environmental water. No amplification was detected from both negative controls; the cooler blank and the equipment blank. The DNA sequences of 8 qPCR products which showed the same haplotype, were identical to the target sequences of Ryukyu ayu (see Suppl. material 2).

The numbers of target DNA fragments (copies/ml) contained in each water sample are listed in Table 1, with standard curve (y = –0.233 + 13.651, R2 = 0.94, efficiency = 71.13%). The average numbers of target DNA fragments detected in each river were as follows: 7.57 ± 7.44 copies/ml (mean ± SD), in the Yakugachi River, 186.52 ± 143.94 copies/ml in the Sumiyou River and 195.6 ± 182.31 copies/ml in the Kawauchi River. The largest number of DNA fragments was detected in segment S3 in Sumiyou River (448.60 copies/ml), and the smallest number was found in segment Y1 in the Yakugachi River (0.72 copies/ml).

**Figure 2.**
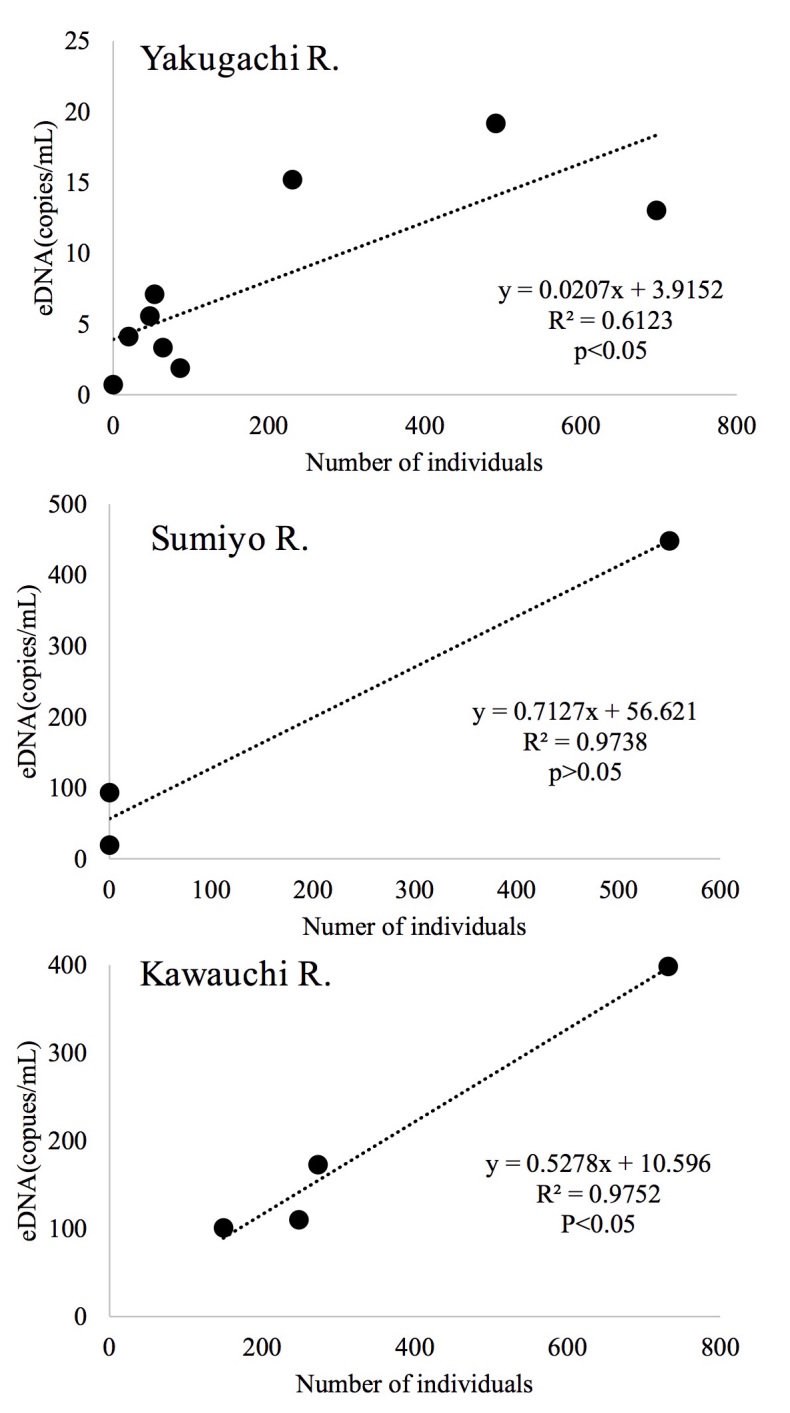
Relationships between the numbers of Ryukyu ayu fish visually counted while snorkelling and the estimated numbers of DNA fragments.

We examined the relationship between individual numbers of fish counted during snorkelling and the number of target DNA fragments in the rivers (Fig. 2). In all rivers, the number of individuals counted was positively correlated with the number of target DNA fragments, particularly in the Yakugachi and Kawauchi rivers (*p* < 0.05; single regression analyses). The coefficients of determination (R^2^) for the relationships were 0.612 (Yakugachi), 0.974 (Sumiyou) and 0.975 (Kawauchi).

## Discussion

We used a non-invasive eDNA procedure to monitor the abundance of Ryukyu ayu. The newly-designed primers for the mtDNA ND4 region (Pa-ND4 primers) amplified target DNA fragments from eDNA samples. Then, we found positive correlations between the individual numbers of Ryukyu ayu counted visually while snorkelling and the number of DNA fragments detected in environmental water samples for all rivers (Fig. 2).

**Figure 3.**
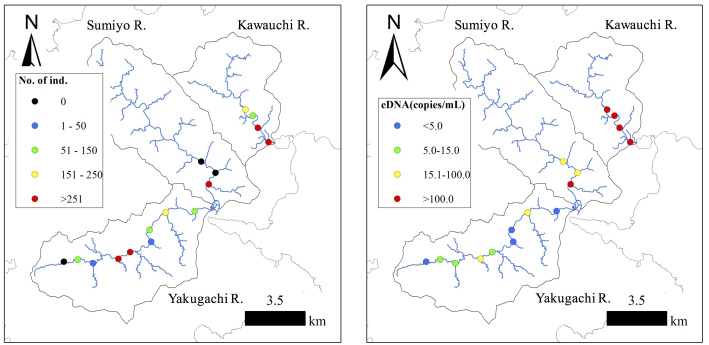
Numbers of individual fish counted visually (left) and numbers of DNA fragments (right) in each segment of the three rivers.

Environmental DNA from Ryukyu ayu was detected in sites where no fish were observed (Table 1, Fig. 3), although these sites were within the known distribution range of Ryukyu ayu (G. Kume, unpublished data). The eDNA densities in these sites were low, possibly originating from only a small number of fish that we failed to detect visually or from eDNA transported downstream. In a previous study on ayu, Doi et al. 2016 also detected eDNA in sites where no fish were observed by visual survey and reported that the molecular approach has considerable promise when fish occur in such low densities that they may not be visually detected. As no negative controls have been amplified by qPCR in this study, the results detecting target species’ eDNA are probably caused by the high sensitivity of this method and not by artificial contamination.

The copy number of detected DNA was smaller in the Yakugachi River than in the other rivers (Table 1). Although the cause of this difference is obscure, we postulate that the concentration of PCR inhibitors was higher in the Yakugachi River than in the other two rivers. Previous studies have shown that the eDNA amplification efficiency is affected by the concentration of PCR inhibitors (potentially algae, polysaccharides and suspended sediment particles) in environmental waters (Klymus et al. 2015, Stoeckle et al. 2017). As another possible cause, eDNA concentration can be strongly affected by characteristics of each river or lotic system, such as water volume, velocity and structures (e.g. riffles and pools) via retention, re-suspension and dilution of eDNA (Shogren et al. 2017). The inconsistency between eDNA concentration and fish abundance in our study may have involved these differences in characteristics of the rivers. While some studies have shown that eDNA analysis methods are applicable to abundance/biomass estimation (Doi et al. 2016, Yamanaka and Minamoto 2016), Buxton et al. (2017), in a study of great-crested newts (*Triturus cristatus*), pointed out that eDNA in aquatic systems is most reliable for detecting presence/absence, but not abundance. Our result implies that the reliable estimation of fish abundance in rivers using eDNA analysis requires *a priori* confirmation of a relationship between the number of eDNA copies and the number of individuals in each river as estimated by independent methods.

## Conclusions

The monitoring of animal distributions and abundances is an essential component of endangered species conservation efforts and stock management of commercial species. However, invasive monitoring methods that use conventional direct trapping negatively affect small populations and are banned for some species, including the Ryukyu ayu. The non-invasive eDNA monitoring method that we used does not contravene current regulations, requires less effort than snorkelling surveys and can, therefore, provide important data.

## Supplementary Material

63B25A71-944F-5D83-AE3F-85FD8A1F778E 10.3897/BDJ.8.e39679.suppl1 Supplementary material 1 Supplimentary file 1 Data type: Alignment file
Brief description: Alignment of each primer/probe sequences with relative species of Plecoglossus altivelis ryukyuensis.
File: oo_342732.txt https://binary.pensoft.net/file/342732 Yoshihisa Akamatsu, Gen Kume, Masuji Goto, Takanori Kono, Takuma Fujii, Ryutei Inui, Yoshihisa Kurita

B3B245E2-6FE3-5AC6-ADC1-093360181EFB 10.3897/BDJ.8.e39679.suppl2 Supplementary material 2 Sequence of eDNA samples Data type: DNA sequence
File: oo_357648.txt https://binary.pensoft.net/file/357648 Yoshihisa Akamatsu, Gen Kume, Masuji Gotou, Takanori Kono, Takuma Fujii, Ryutei Inui, Yoshihisa Kurita

## Figures and Tables

**Table 1.** Individual numbers of the Ryukyu ayu and eDNA amplification efficiency.

River	Site	Number of Ryukyu ayu	Detection rate (detected well number/total well number)	Average number of detected DNA fragments (copies/ml) ± SD
Yakukachi River	Y1	0	2/4	0.72 ± 0.83
	Y2	53	4/4	7.11 ± 8.32
	Y3	47	4/4	5.58 ± 2.51
	Y4	491	4/4	19.19 ± 6.67
	Y5	697	4/4	13.03 ± 3.84
	Y6	20	4/4	4.12 ± 2.74
	Y7	64	3/4	3.34 ± 2.45
	Y8	230	4/4	15.21 ± 5.41
	Y9	86	4/4	1.89 ± 1.05
Sumiyo River	S1	0	4/4	19.50 ± 28.22
	S2	0	4/4	93.74 ± 9.81
	S3	550	4/4	448.60 ± 38.84
Kawauchi River	K1	248	4/4	110.30 ± 80.63
	K2	149	4/4	100.90 ± 55.20
	K3	732	4/4	398.40 ± 264.53
	K4	273	4/4	172.77 ± 97.32
